# The Heritability of Shell Morphometrics in the Freshwater Pulmonate Gastropod *Physa*

**DOI:** 10.1371/journal.pone.0121962

**Published:** 2015-04-13

**Authors:** Robert T. Dillon, Stephen J. Jacquemin

**Affiliations:** 1 Department of Biology, College of Charleston, Charleston, South Carolina, United States of America; 2 Department of Biology, Wright State University—Lake Campus, Celina, Ohio, United States of America; University of South Carolina, UNITED STATES

## Abstract

The cosmopolitan freshwater pulmonate snail *Physa acuta* hybridizes readily with *Physa carolinae* in the laboratory, although their F1 progeny are sterile. The two species differ qualitatively in shell shape, the former bearing a more globose shell and the latter more fusiform. We performed a hybridization experiment, measuring a set of 14 traditional (linear) and landmark-based shell morphological variables on even-aged parents and their offspring from both hybrids and purebred control lines. Parent-offspring regression yielded a strikingly high heritability estimate for score on the first relative warp axis, h^2^ = 0.819 ± 0.073, a result that would seem to confirm the value of geometric morphometrics as a tool for retrieving evolutionary relationships from gastropod shell form. Score on the second relative warp axis was also significantly heritable (h^2^ = 0.312 ± 0.123), although more moderate, as were scores on second principal components extracted from traditional measurements (correlation h^2^ = 0.308 ± 0.069, covariance h^2^ = 0.314 ± 0.050). Although score on the first relative warp axis was significantly correlated with centroid size (p < 0.001), scores on none of the three second axes were so correlated. This result suggests that second axis score might prove especially useful for estimating genetic divergence among mixed-age populations of gastropods sampled from the field.

## Introduction

The methods by which the biological information conveyed in gastropod shell morphology have been retrieved and analyzed are as diverse as the origins of that information itself. The collection of simple linear measurements, such as shell length and shell width, has been a standard of malacological practice since the nineteenth century. Theoreticians [1] have explored modeling approaches to compare the vast diversity of gastropod shell form naturally observed to that which might be possible. With the advent of multivariate morphometrics in the 1970s [2,3], evolutionary biologists began analyzing gastropod shell morphology with principal components [4], discriminant functions [5,6], and factor analysis [7].

In the early 1990s, the study of morphometrics was revolutionized by the development of new analytical methods designed to capture the geometrical relationships among sets of digitized landmarks [8]. The first applications of geometric morphometric techniques to gastropod shells were the studies of Johnston et al. [9] on three populations of the marine intertidal snail *Epitonium*, the research of Wagner [10,11] on Paleozoic fossils, and the simulations of Stone [12].

Regardless of methodological detail, however, the primary motivation for most of the studies cited above has been to use phenotypic variance in shell morphology as an estimate of genetic relationships among sets of natural gastropod populations. At least two challenges have long been apparent. One obvious hurdle is that gastropod populations sampled from the wild are typically composed of mixed-age individuals demonstrating indeterminate growth. Thus some large fraction of the total variance in shell phenotype manifest by most natural populations is not expected to be heritable, but simply to arise as a function of the mixture of ages in the sample.

For this reason, malacologists of the 1970s and 1980s were attracted to principal component analysis. Most of the variance in linear shell measurements is expected to load uniformly and positively onto the first principal component, which can then be taken as a measure of overall size, and set aside [13]. This challenge has been addressed differently in more recent geometric morphometric practice, size being effectively subtracted by superimposition [14].

A second hurdle is the problem of ecophenotypic plasticity. In many gastropod populations, some significant fraction of the variance in shell morphology is not additively heritable, but rather seems to arise as a direct phenotypic response to the local environment. In the intertidal dog whelk *Acanthina monodon*, for example, populations exposed to wave action form shells with lower spires and rounded apertures, while those inhabiting sheltered environments bear shells with higher spires and narrow apertures. But the shell morphology of pre-hatching juveniles does not differ between sheltered and exposed populations, and heritability estimates from the analysis of sibling groups suggest that a large fraction of the variance in shell morphology observed in adults is a plastic response to wave action [15].

The potential for ecophenotypic plasticity in freshwater gastropod populations seems to be especially high. Evidence of direct effects of current and flow upon shell morphology in the pulmonate snail *Lymnaea* (or *Radix*) has been offered by Lam and Calow [16], effects of water chemistry have been demonstrated by Rundle et al. [17], and predator effects have been documented by Bronmark et al. [18,19]. Evidence of ecophenotypic responses in shell morphology to predator pressure have also been documented in populations of the pulmonate planorbid *Helisoma* [20,21] and in the prosobranch pleurocerids [22–24]. Rearing morphologically diverse populations in a constant environment (“common garden experiments”) suggest a significant ecophenotypic component to shell height in the pulmonate limpet *Ferrissia* [25].

In recent years a growing community of researchers has been attracted to freshwater pulmonate snails of the genus *Physa* as a model organism for the study of behavior, sex allocation, divergence and speciation [26–28]. The cosmopolitan *Physa (“Physella”) acuta* in particular has become a favorite for laboratory experimentation, by virtue of its ease of culture, rapid generation time, and individual lifetime fecundity ranging into the thousands of offspring [29,30]. Laboratory populations of *Physa acuta* (under a variety of synonyms) have been shown to demonstrate a significant ecophenotypic response in shell shape both to temperature [31] and to the introduction of crushing predators [32–35]. The common garden experiments of Gustafson et al. [36] returned evidence of striking morphological plasticity in the shell form of *P*. *acuta* correlated with the (many) environmental differences between stream and pond.

Less well known is *Physa carolinae*, an inhabitant of vernal ponds and ditches in the Atlantic coastal plain of the southeastern United States. *Physa carolinae* and *P*. *acuta* hybridize readily in the laboratory, although hybrid offspring appear to be entirely sterile [37]. In the field, the two species are distinguishable by minor aspects of shell shape. *Physa carolinae* populations tend to bear more slender, fusiform shells while *P*. *acuta* bear shells more inflated, globose [38].

What fraction of the variance in shell morphology qualitatively observable in a comparison of *Physa acuta* and *P*. *carolinae* might be additively heritable? What analytical techniques might maximize the heritable fraction recovered? Are modern geometric approaches so superior to more traditional linear methods that all previous research results are now discredited? Here we report the results of *acuta* x *carolinae* hybridization experiments, analyzing shell morphological data collected on sets of even-aged parents and offspring with both traditional and landmark-based techniques. We evaluate a large set of univariate and multivariate shell variables by two criteria—their estimated heritability and their correlation with overall size. In so doing we look toward future applications of shell morphometric analytical techniques in mixed-age gastropod populations collected from the wild. We also offer our results to the community of researchers interested in the reconstruction of gastropod phylogeny [39], where the heritability of shell form is an oft-unstated assumption.

## Materials and Methods

Our standard methods for *Physa* culture, involving plastic 10 ounce (220 ml) drinking cups of filtered pond water with Petri dish lids and *Spirulina*-based flake fish food, have been described by Wethington and Dillon [27, 40]. Adult snails were isolated from two stock cultures, the *Physa acuta* line-15 albinos of Dillon and Wethington [41,42] and the “Julian” line of *Physa carolinae*, from the Dill Wildlife Refuge on James Island, within the metropolitan Charleston area. Both of these laboratory lines were established in the early 1990s, for which no specific permissions were required. Egg masses were accumulated over several days, allowed to hatch, and a sample of 1–2 mm hatchlings themselves isolated at age two weeks, well in advance of maturity [29]. Isolated juveniles from the two lines were reared an additional 17 weeks, by which time most had begun to reproduce by self-fertilization [29]. Twelve virgin *P*. *acuta* and 12 virgin *P*. *carolinae*, demonstrating successful self-fertilization, were then arbitrarily selected to serve as the parental generation for our experiment.

Four pairs of parental snails were crossed within the *acuta* line, four pairs crossed within the *carolinae* line, and four pairs were hybridized. These 12 sets of parents were paired for 24 hours, re-isolated for six days, and their egg masses accumulated. The parents were then sacrificed whole (by preservation in 70% ethanol) at age 20 weeks.

Egg masses of the F1 generation were allowed to hatch and the offspring reared to age two weeks. The F1 juveniles produced by the albino (*acuta*) parent of the *acuta* x *carolinae* outcross proved to be pigmented in all cases, confirming their hybrid status. We do not have direct confirmation that the offspring of the *acuta* x *acuta* and *carolinae* x *carolinae* controls were the products of outcross, but previous breeding experiments aided by genetic markers have demonstrated an almost 100% shift from selfing to outcrossing in similar situations [29,40]. Then five haphazardly-chosen F1 individuals from each of the 12 sibships were isolated, reared to age 20 weeks, and sacrificed whole by preservation in 70% ethanol.

### Traditional morphometrics

**Fig 1:**
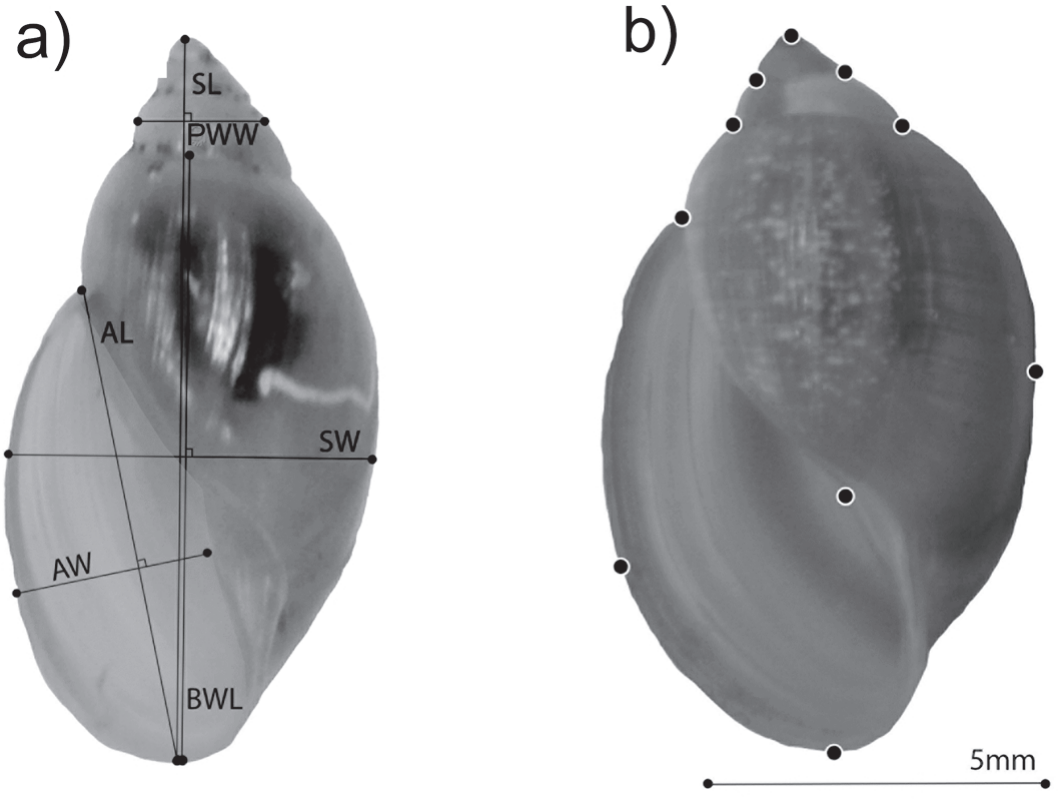
Example shells from (a) *Physa carolinae* and (b) *Physa acuta*. The six linear measurements are shown on shell a: Shell length (SL), shell width (SW), body whorl length (BWL), penultimate whorl width (PWW), aperture length (AL) and aperture width (AW). The 11 landmarks are located on shell b. Landmark points follow Dunithan et al. [47] and include points to capture the apex, aperture, lip, body whorl, and spire whorls. Landmarks on main body whorl and aperture lip were treated as sliding semilandmarks.

The shells of the 12 pairs of parents and their 60 F1 offspring were positioned in a standard format and photographed adjacent to a scale bar using a mounted Nikon d3000 camera with Nikkor 60mm lens. Six simple linear measurements were made on each image using tpsDIG [43]: shell length, shell width, aperture length, aperture width, body whorl length, and penultimate whorl width (Fig. 1A). Then PC-ORD [44] was used to extract principal components from both the correlation and covariance matrices of the linear measurements. The broken stick model [45], which has been shown to perform well with correlated variables such as morphological attributes, was used to assess axis significance prior to interpretation.

### Geometric morphometrics

The tpsDIG application of Rohlf [43] was also applied to describe all 84 shells with 11 fixed or repeatable (homologous) landmarks using standard geometric morphometric techniques [46]. Landmark points followed Dunithan et al. [47] and included points to capture the apex, aperture, lip, body whorl, and spire whorls (Fig. 1B). To more closely approximate the curvature of the main body whorl and aperture lip, two landmarks were treated as sliding semilandmarks, positioned medial relative to fixed landmarks on edges where no fixed points were readily identifiable [46].

Centroid size was calculated for each of the 84 shells as the square root of the sum of the squared distances from each of the 11 landmarks to their joint median using tpsRELW [48]. Simple correlation with centroid size was used as an estimate of the potential importance of body size [49] to variance in all of the other (ultimately 13) morphometric variables using the R statistical environment [50]. The increased likelihood of Type I error inherent in interpretation of the multiple p-values that result from such comparisons was corrected using Q-VALUE version 1.38.0 [51,52]. The q-value approach is a false discovery rate technique that improves over (more conservative) Bonferroni-type corrections by using the distribution of p values to assess the proportion of false positives.

Procrustes techniques were applied to remove the effects of translation, rotation, and scaling in our landmark data [46]. Then morphometric shape variation was described using relative warp analysis (i.e. principal components analysis of landmark data) and visualized using thin-plate spline methods with tpsRELW [48]. Resulting morphometric landmark axes were interpreted using percent variation explained and individual landmark loading scores as visualized by deformation grids.

### Estimating heritability

The (narrow sense) heritability of a trait (symbolized h^2^) is the proportion of its total phenotypic variance that is “additive,” available for selection to act upon. Among the most direct methods by which this statistic can be estimated is parent-offspring regression [53,54]. Where the phenotypic scores for some trait of interest are measured on sets of offspring (“sibships”), and plotted as a function of the average scores of their parents (the “midparent value”), the slope of the regression line is an estimate of the narrow-sense heritability of the trait. Normality of the underlying variance structure is assumed. Thus when heritability is estimated across populations, hybrids as well as inbred pairings must be analyzable.

We estimated the narrow-sense heritability for each of the six linear measurements, the PCA scores (on both correlation and covariance matrices), the RWA scores, and centroid size using simple linear regression of offspring values on midparent values across the 12 sibships in the R statistical environment [50]. The q-value method was again employed to estimate the significance of the (now 14) independent sets of regression statistics.

## Results

Summary statistics for the six simple linear measurements taken on our sample of 84 snails (parents and offspring combined) are reported in Table 1. The first principal component (PC) extracted from the correlation matrix of these six variables across the 84 measurements accounted for 92.1% of the variance, all variables loading positively and evenly, as expected for such data. The second PC accounted for but 5.5% of the variance, with penultimate whorl width demonstrating an especially high positive loading, shell width and aperture width exhibiting negative axis loadings. Extracted from the covariance matrix, the first principal component accounted for 96.5% of the variance, all factor loadings again positive, although weighted toward the variables with the highest variance: shell length and body whorl length. The second covariance PC (accounting for but 2.4% of the variance) demonstrated positive loadings on aperture length, aperture width and shell width, with negative loadings on the remaining variables. Summary statistics for factor scores on both principal components extracted from both the correlation and covariance matrices across all 84 observations are also reported in Table 1.

**Table 1:** Summary statistics for shell morphology in lab reared populations of *Physa* at age 20 weeks, 24 parents and 60 offspring combined.

**Variable**	**Mean**	**S.D.**	**Min.**	**Max.**	**Correlation**	**q-value**
Shell length (mm)	77.5	14.15	50.0	115.0	0.992	0.001
Shell width (mm)	43.6	7.28	30.0	63.0	0.936	0.001
Aperture length (mm)	57.4	9.49	41.0	82.0	0.959	0.001
Aperture width (mm)	24.7	4.33	18.0	35.0	0.917	0.001
Body whorl length (mm)	67.0	11.42	45.0	93.0	0.992	0.001
Penult. whorl width (mm)	14.6	3.05	9.0	24.0	0.910	0.001
PC1 (correlation)	0	2.35	-5.705	4.313	0.986	0.001
PC2 (correlation)	0	0.5744	-1.1829	1.598	0.261	0.053
PC1 (covariance)	0	0.22	-0.5194	0.4192	0.989	0.001
PC2 (covariance)	0	0.03457	-0.0963	0.0769	0.263	0.053
RWA1	0	0.03533	-0.0723	0.0815	-0.385	0.001
RWA2	0	0.03217	-0.0517	0.0799	-0.133	0.228
RWA3	0	0.02317	-0.0507	0.0685	0.139	0.226
Centroid size (mm)	0.9433	0.1653	0.6201	1.3794	1.000	-

The correlation is with centroid size, significance estimated by q-value [51], adjusted for N = 13 comparisons.

**Fig 2:**
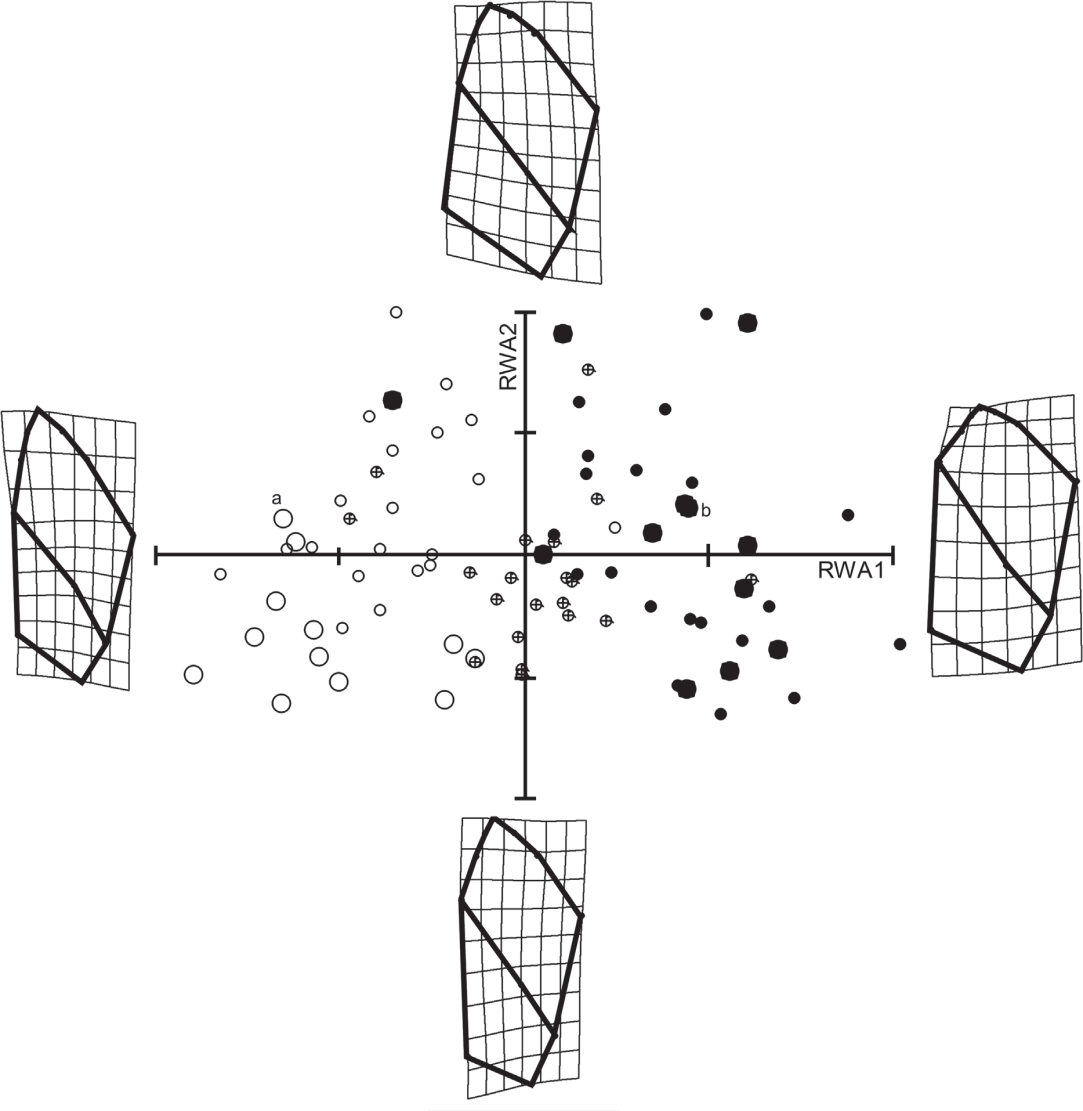
The 84 shells plotted on Relative Warp Axes 1 and 2. *Physa carolinae* are shown as open circles, *Physa acuta* as filled circles, and hybrids as cross-circles. The parental generation is plotted as large symbols, and their F1 offspring as small symbols. Letters a and b indicate the two shells depicted in Fig. 1.

Principal components analysis of aligned landmark coordinates (“relative warp analysis”) for our sample of 84 shells yielded 12 axes accounting for greater than 1% of the variance each, together explaining a total of 97.2% of the variation among individuals. Relative warp axis 1 explained 29.7%, RWA2 explained 24.6% and RWA3 explained 12.8%. Fig. 2 shows that variance on RWA1 was associated with shell slenderness and spire height, such that shells scoring high on axis 1 were more globose, and those scoring lower more fusiform. Variance on RWA2 seemed most associated with aperture shape, shells with higher scores demonstrating broader and taller apertures as well as larger aperture opening area. RWA3 (not shown) was also associated with increased aperture opening area. Summary statistics for scores on the first three relative warp axes across the combined sample of 84 shells are given in Table 1.

The bottom row of Table 1 reports summary statistics for centroid size. The correlation between centroid size and each of the 6 simple linear measurements, the four PCA scores, and the three RWA scores is shown in the rightmost column of Table 1. Almost all of these 13 variables proved highly correlated with centroid size. The exceptions were both second principal components from the analyses of linear measurement matrices (correlation and covariance), which were barely nonsignificant (q = 0.053), and RWA2 and RWA3, which were apparently uncorrelated with size.

Statistics for the regressions of the 60 offspring on their 12 midparent values across all 14 morphological variables are reported in Table 2. Half of the 14 variables appeared to demonstrate significant heritabilities: two of the simple linear measurements, two sets of principal component scores, two sets of relative warp scores, and centroid size. To place the results shown in Table 2 in a broader context, livestock researchers conventionally characterize h^2^ < 0.2 as low heritability, 0.2 > h^2^ < 0.4 as moderate heritability, and h^2^ > 0.4 as high heritability [55].

**Table 2:** Statistics from parent-offspring regressions.

**Variable**	**Heritability (± s.e.)**	**R** ^2^	**F**	**q-value**
Shell length	0.429 (± 0.139)	0.142	9.57	0.008
Shell width	0.030 (± 0.110)	0.001	0.07	0.845
Aperture length	0.023 (± 0.145)	0.000	0.03	0.875
Aperture width	0.055 (± 0.148)	0.002	0.14	0.832
Body whorl length	0.321 (± 0.147)	0.076	4.78	0.058
Penult. whorl width	0.401 (± 0.121)	0.159	10.98	0.007
PC1 (correlation)	0.157 (± 0.149)	0.019	1.11	0.377
PC2 (correlation)	0.308 (± 0.069)	0.253	19.68	0.005
PC1 (covariance)	0.253 (± 0.148)	0.048	2.94	0.143
PC2 (covariance)	0.314 (± 0.050)	0.404	39.35	0.005
RWA1	0.819 (± 0.073)	0.685	126.0	0.005
RWA2	0.312 (± 0.123)	0.102	6.58	0.026
RWA3	-0.618 (± 0.464)	0.030	1.78	0.263
Centroid size	0.384 (± 0.144)	0.110	7.15	0.023

Significance of the regression is estimated by q-value [51], adjusted for N = 14 comparisons.

**Fig 3:**
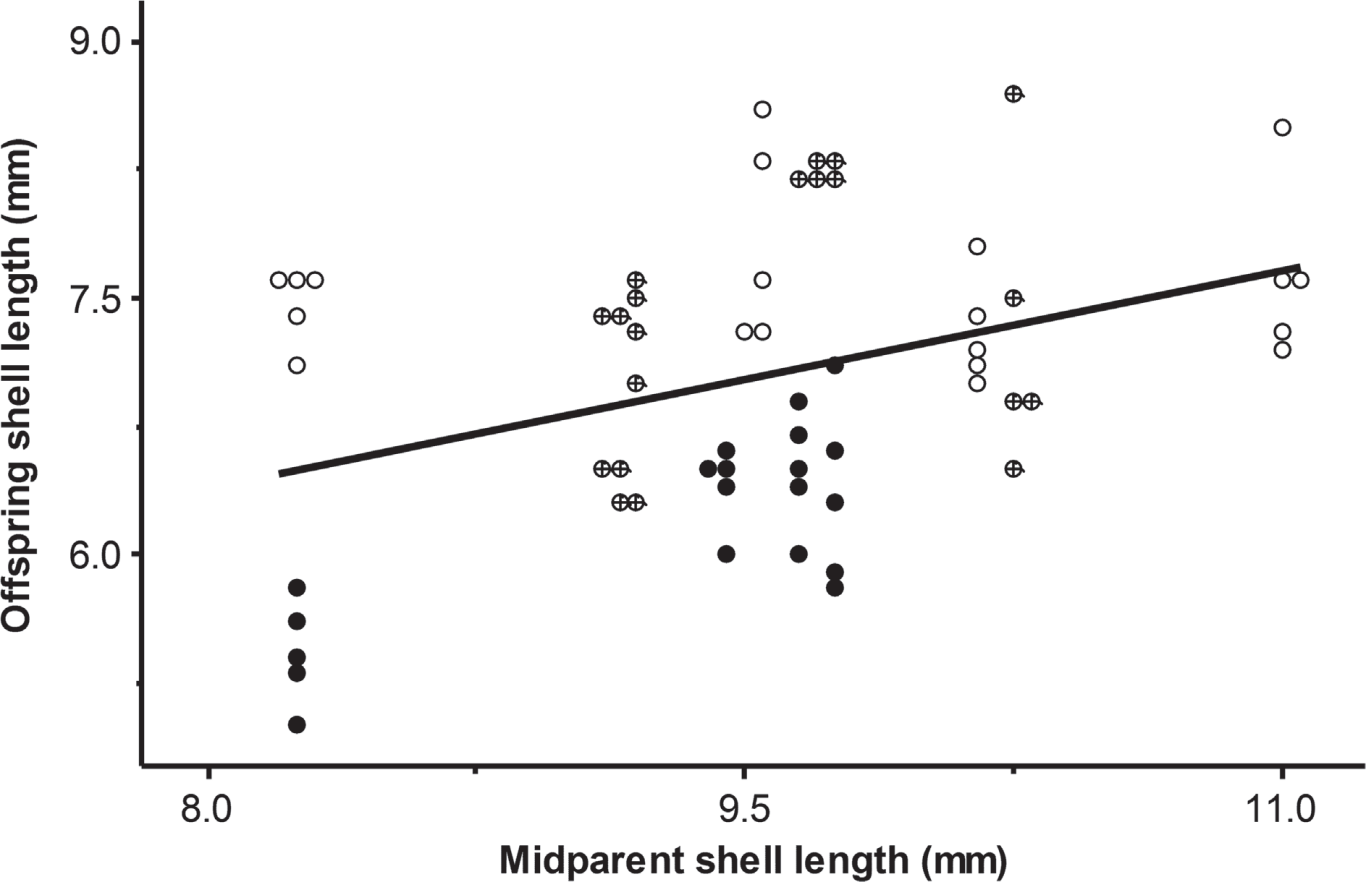
Parent-offspring regression demonstrating the relationship y = 0.429x + 0.295 for simple shell length (SL). *Physa carolinae* are plotted as open circles, *Physa acuta* are filled circles, and hybrids are cross-circles.

Then two of the six simple linear measurements appeared to demonstrate high heritability: shell length and penultimate whorl width. Inspection of the graphic results for shell length did not suggest that hybrids were intermediate in their shell morphology between the pure lines (Fig. 3), although there was some evidence of such a tendency for penultimate whorl width.

Parent-offspring regressions returned strikingly similar heritability estimates for factor scores from both principal component analyses based on simple linear measurements—correlation and covariance. While factor scores on neither first principal component demonstrated significant heritability, the heritabilities of both second principal components were moderate and significant. And inspection of graphic results for both second principal components showed the four hybrid sibships demonstrating factor scores intermediate between the factor scores of the two purebred lines.

**Fig 4:**
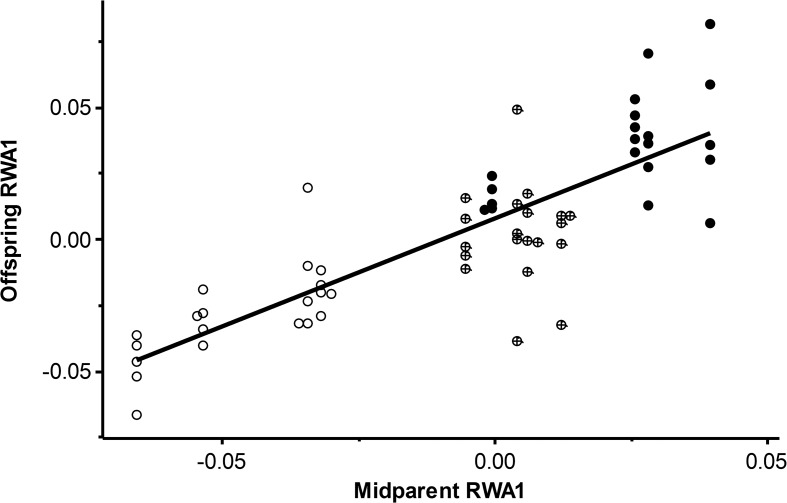
Parent-offspring regression demonstrating the relationship y = 0.819x + 0.008 for score on Relative Warp Axis 1 (RWA1). *Physa carolinae* are plotted as open circles, *Physa acuta* are filled circles, and hybrids are cross-circles.

Regression of offspring RWA1 score on midparent RWA1 value returned a strikingly high heritability estimate of 0.819 ± 0.073 (Fig. 4). The heritability of relative warp axis 2 score was also significant, although moderate, proving comparable in this respect to the heritability demonstrated by the second principal components extracted from the matrices of traditional linear measurements. And indeed the heritability of overall centroid size was also in this same range, statistically significant although biologically moderate.

## Discussion

The published literature contains a wide variety of estimates for the heritability of gastropod shell morphological variables, as might be predicted by the diversity of the Class Gastropoda, and the diversity of the methodologies employed. The review of Dupont-Nivet et al. [56] returned seven older studies of heritability for simple linear shell metrics in five species of land snails, ranging from h^2^ = 0.16 ± 0.06 to 0.81 ± 0.42. The authors themselves used the regression of lab-reared offspring on wild-collected parents to obtain an estimate of h^2^ = 0.36 ± 0.16 for shell diameter in the land snail *Helix aspersa*. Their estimate for the heritability of this same trait using analysis of variance among (entirely lab-reared) sibships was h^2^ = 0.68 ± 0.12.

More recent research interests seem to have turned toward the heritability of shell morphology in the gastropod fauna of the rocky intertidal marine environment. Boulding and Hay [57] reared full-sib broods of littorinid periwinkles, using three analytical methods to obtain estimates for the heritability of eight shell-shape ratios and principal components, ultimately reporting 18 values ranging from nonsignificance up to h^2^ = 0.55 (error unspecified). Carballo et al. [58] estimated heritabilities for a set of 12 linear measurements and ratios using both the regression of lab-reared offspring on wild-collected mothers and full-sib correlation in seven samples of *Littorina*; the 7 x 12 x 2 = 168 values obtained ranging from nonsignificance to h^2^ = 0.717 (error unspecified). Conde-Padín et al. [59] used full-sib correlation to estimate the heritabilities of five landmark-based multivariate traits in three populations of littorinids, the 5 x 3 = 15 values ranging from h^2^ = 0.363 ± 0.059 to 0.941 (error unspecified) for the first relative warp axis in a laboratory-bred set of full-sib juveniles. In the muricid dogwhelks, Guerra-Varela et al. [60] used full-sib correlations to obtain heritability estimates for 7 landmark-based shell traits in two wild-collected populations of *Nucella*, ranging from nonsignificance to h^2^ = 0.56 ± 0.218. Solas et al. [15] used similar methodologies to estimate the heritabilities of five landmark-based shell variables in six *Acanthina* populations, the values obtained ranging from nonsignificance to h^2^ = 0.47 ± 0.15. Lucas et al. [61] estimated h^2^ = 0.48 ± 0.15 for shell length and 0.38 ± 0.13 for shell width in a mass culture of the abalone *Haliotis asinina*, using microsatellite markers to assign parentage.

Compared with studies conducted in the marine and terrestrial environments, the published research on heritability of shell morphology in populations of freshwater gastropods is very sparse. The mother-offspring regressions of Urabe [62] returned negligible heritabilities for three shell-shape ratios in the pleurocerid *Semisulcospira*. Chaves-Campos et al. [63] used microsatellite markers to reconstruct genetic relationships in a sample of wild-collected hydrobiid snails, using two methods to obtain two estimates for both landmark-based and PC2-based measures of shell shape, the 2 x 2 x 2 = 8 values obtained ranging from nonsignificance to h^2^ = 0.30 (error unspecified).

The heritability shown in Fig. 4 for RWA1 score in the freshwater pulmonate *Physa*, h^2^ = 0.819 ± 0.073, is among the highest heritabilities reported for any shell morphological variable in any gastropod population to date. Indeed, with 68.5% of the variance explained by the regression, little variance would seem residual for measurement error, much less for an environmental component. This finding constitutes a strong endorsement of landmark-based geometric morphometrics as a tool for retrieving the genetic relationships among gastropod populations, particularly when reared under controlled conditions.

Variance on the first relative warp axis comprised only 29.7% of the total, however. Perhaps the most interesting contrast shown in Table 2 is that between our RWA1 results and the results from the first principal components extracted from matrices of traditional linear shell measurements, both accounting for over 90% of the total variance recovered, but neither demonstrating significant heritability. The observations that all three of these first axes—RWA1, PC1 (correlation), and PC1 (covariance)—were correlated (p < 0.001) with centroid size, and that RWA1 demonstrated one of the highest heritabilities yet recorded for gastropod shell morphology, while neither of the first principal components from traditional linear measurements demonstrated any heritable basis whatsoever, might seem a bit paradoxical. Apparently our traditional morphometric approaches lumped together a very large fraction of the total variance in shell morphology as “size,” while our geometric approach identified and isolated a subset of such variance that might be described as “heritable size.” Fig. 4 suggests that our pure *acuta* x *acuta* families were heritably larger than our pure *carolinae* x *carolinae* families, with hybrid families intermediate.

The efficiency by which geometric morphometric techniques eliminate size while preserving allometric relationships is well established [46,64]. Thus the eye also detects shape components to both of the axes graphed in Fig. 2, shells scoring high on RWA1 apparently broader and more inflated than shells scoring low. It seems likely to us that the preservation of allometric variance on RWA1 may largely explain its strikingly high heritability, and might point toward the utility of landmark-based morphometrics in life history studies of freshwater gastropod populations more generally.

The other interesting result to emerge from inspection of Table 2 is the similarity of the heritabilities of all three of our second axes. The second PC from the correlation matrix (5.5% of the variance) showed h^2^ = 0.308 ± 0.069, the second PC from the covariance matrix (2.4%) showed h^2^ = 0.314 ± 0.050, and RWA2 (24.6%) showed h^2^ = 0.312 ± 0.123. Variance on none of these axes was significantly correlated with centroid size. Hence the genetic component of this variance would not seem as potentially sensitive to the assumption of constant age.

Heritabilities estimated in the laboratory can be surprisingly reflective of heritabilities in nature [65,66]. Thus variance on the second axis of a shell morphometric analysis, regardless of how extracted, might (with caution) be used to estimate genetic relationships among mixed-age gastropod populations sampled from the field. The practice (widespread in the 1970s and 1980s) of using second principal component scores for this purpose here receives some justification. But again, preference might be accorded to geometric techniques, since the variance in RWA2 was substantially greater than the variance in the second principal components from traditional measurements, and there was less evidence of a correlation with size.

We offer three caveats, however. First, the present analysis has focused upon interspecific variance, which we had some prior reason to expect might be heritable in its origin. By extending our parent-offspring regression across a pair of reproductively isolated species and their hybrids, we consciously increased the total phenotypic variance (presumably including genetic variance) relative to the measurement error, pushing our estimates of heritability upward.

Second, the estimates of heritability offered in Table 2 depend on the normality of the shell morphological variance underlying our entire experiment, across both species combined. There is certainly reason the fear that this assumption may not hold in our case, although the consequences of its violation are difficult to predict. Note that assortative mating designs such as we have employed here are a standard approach to increase the precision of heritability estimates, however, and do not (by themselves) introduce bias [53].

Third, the potential for ecophenotypic plasticity to confound an analysis such as ours seems to be especially acute in gastropod populations sampled from natural fresh waters. We are aware of at least 35 studies published since 1982 specifically focusing upon the environmental component of shell morphological variance in freshwater gastropod populations sampled from the wild, only a small fraction of which was touched upon in our introduction. Against such a background, however, the results reported in the present work can be viewed as providing a small measure of balance.
